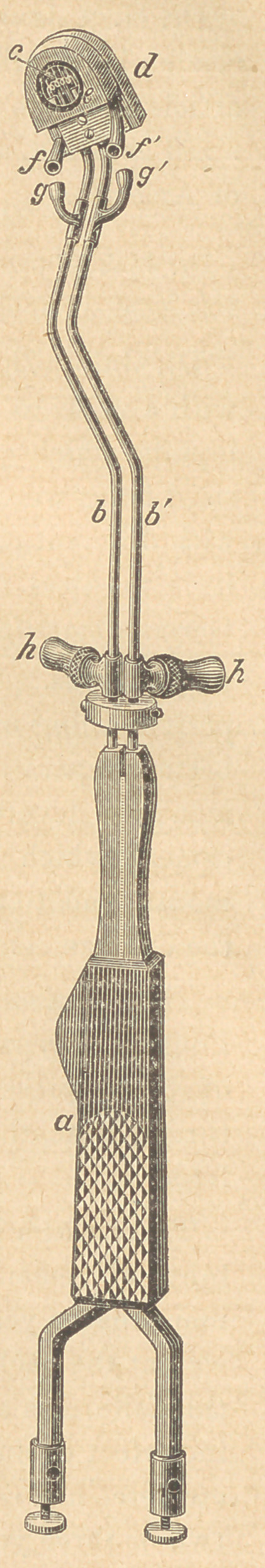# Report of the Meeting of the American Dental Society of Europe

**Published:** 1883-11

**Authors:** W. D. Miller

**Affiliations:** Berlin


### REPORT OF THE MEETING OF THE AMERICAN DENTAL SOCIETY OF EUROPE, HELD AT COLOGNE, GERMANY.

BY DR. W. D. MILLER, OF BERLIN.

The eleventh annual meeting of the American Dental Society of Europe was held at the Grand Hotel Victoria, in Cologne, on the 7th, 8th and 9th of August, 1883.

Dr. N. S. Jenkins, of Dresden, opened the regular work of the session by a serio-comical essay entitled "A Day's Practice." This article gave the methods daily used in practice by one who is recognized as being, as a preserver of teeth, inferior to no one in Europe or America.

Dr. Jenkins' paper will appear in the December number of THE INDEPENDENT PRACTITIONER.

Dr. Galbraith, of Dresden, described a series of experiments made with a view to determining the solubility of various cements in liquids, which are known to occur in the human mouth. Equal sized holes were bored in strips of hard wood ; those were filled with different cements, and the strips submerged in various liquids ; it is needless to say that the pyrophosphates, especially those of German manufacture, showed a decided advantage over all other preparations. While the American has long looked upon gold as a perfect filling material, and has directed all his efforts to securing better preparations and better methods of manipulating it, the German has devoted himself with equal zeal to the production of a cement which should be insoluble in the liquids of the human mouth, and to this fact, no doubt, is due the evident superiority of the German cements.

The general experience of the society was to the effect that Poulson's preparations were the best in use ; it was also found that of those present seventeen preferred Poulson, seven Rostaigne, three Frieze, three Worff, two Weston, two Fossiline, and one Eisfelder.

There was a great variety of opinion as to the value and durability of cements, some not allowing them on approximal surfaces more than a year's existence, having found them invariably to give way at the neck of the tooth in a few months ; others looked upon this failure as a result of imperfect manipulation of the material, imperfect exclusion of moisture, too hasty insertion and polishing, etc., etc., and considered a properly inserted cement filling good for two or three years at least.

Dr. Jenkins exhibited an apparatus for illuminating the oral cavity, devised by Dr. Josef Leiter, of Vienna. (See figure.) By means of two insulated copper wires, set in the handle, *a*, and the two metallic tubes *b* and *b′*, a galvanic current is conducted through the platinum spiral *c*. The tubes *b g* and *b′ g′* serve also for conducting a stream of water,and when *g* and *g′* are connected with *f* and *f′* by short rubber tubes, a continuous current of fresh water may be conducted through *h*, *b*, *g*, *f*, *d*, *f′ g′*, *b′*, *h′*, which absorbs the heat produced by the glowing platinum wire beneath the glass plate *c*, thereby preventing the instrument from becoming uncomfortably warm. This apparatus, though somewhat complicated, may be used in the mouth with wonderful effect.

An apparatus of the same character, known as the "Frontal Electric Photophone," was exhibited by Dr. J. W. Crane, of Paris, who gave the following description :—"This ingenious appliance, the invention of the well-known electrician, Monsieur G. Trouve, is a welcome application of the electric lamp in medicine as well as in domestic use. Hitherto it has only been possible to employ the electric lamp on a large scale, by obtaining power from powerful dynamo or magneto-electric machines. Monsieur Trouve has found that this light may be utilized in every household at a comparatively small expense.

Dr. Paul Hélot, chief surgeon of the hospitals of Rouen, and Monsieur Trouve, have solved in a no less satisfactory manner the problem of the electric light in surgery. The apparatus is composed of a Swan lamp, held in a metallic cylinder between a reflector, an objective, and two conducting wires.

This remarkable improvement is very light and small, and is fastened on the forehead like the mirror of the laryngoscopist or of the aurist. The light which it supplies is very intense, but can be modified according to necessity by the addition or suppression of one or more cells in the battery. The full force of six cells on one lamp would be too powerful for the extremely delicate carbons of the lamp.

The rays of light are directed by a slight alteration of the objective, with the greatest facility.

Placed in the axis of vision, the light accompanies the sight of the operator, both sight and light being brought to a focus together, the operator himself having no further anxiety about it.

In case one prefers to employ the lamp without applying it to the forehead, nothing could be more simple than to fasten it on a support, on a table, on the arm of the chair, or upon any piece of furniture whatever.

The supply of power is the pile, saturated with bichromate of potash, invented by M. Trouve.

It can, in this case, be used a great number of hours without being recharged, either continuously or at intervals, as long as required.

This lamp for lighting can be applied in a great number of cases; it may serve to cast light in deeply situated cavities, such as the mouth, the throat, the ears, etc. The gynecologists will frequently find it useful in certain delicate operations demanding much light, but it is above all in diseases of the throat and ears that its use seems preferable to all other apparatus, as the light which it supplies is perfectly white, and does not alter the color of the tissues. Henceforth dentistry will not only demand electric power for the dental engine by means of the pile, but without perceptibly increasing the expense we will have the means of lighting the mouth by the use of the photophone in a way that leaves nothing to be desired. To sum up, the apparatus is a useful discovery, is made with precision by the intelligent constructor, and seems destined to render real service to most medical men."

Dr. Rosenthal of Liege read an article on the "Antiseptic Treatment of the Dental Pulp," giving a resume of Witzel's methods. (See page 585).

DISCUSSION.

*Dr. Kellner.*—I have for years been making experiments in the antiseptic treatment of the dental pulp, and my experience teaches me that the amputation of exposed pulps is followed in most cases by bad results, as the remaining parts of the pulp die in the course of time, giving rise to inflammation of the periodontium, frequently necessitating the extraction of the tooth. My friend, Dr. Witzel, writes me that he has, even by the utmost care in amputating the pulp, had a number of bad results.

*Dr. De Trey.*—Those of you who were present at the meeting of our society at Ostende, will perhaps remember a paper which I presented on the "Resection and Cicatrization of the Dental Pulp." I have made use of the methods advocated in that paper with continued success, having in the course of two years had but one failure. Let us suppose that we have an upper molar whose pulp is exposed at the anterior buccal horn. I immediately apply the rubber dam, as the subsequent operation is thereby very much facilitated, and perfect cleanliness secured, which is absolutely essential to the success of the operation. After clearing the cavity of all rubbish and decayed tissue with instruments particularly constructed for the purpose, I cut a groove around the point of exposure so that the latter is left standing out as a little mound from the bottom of the cavity; then with one stroke of a properly shaped excavator the protruding hill of dentine is removed along with the inflamed portion of the pulp. The cut must be clear and clean, not breaking the dentine or tearing the pulp; it then produces very little pain and the wound heals very rapidly. Any pieces of dentine which may be forced into the tissue of the pulp must be carefully removed. The pulp is allowed to bleed freely, nothing else than tepid water being used to rinse out the cavity, and when the bleeding has thoroughly ceased I absorb the excess of moisture from the surface by means of punk which is free from nitrate of potassium, and fill immediately over the pulp with Worff's oxychloride of zinc. Some pain occurs as a rule after introducing the filling, which however subsides in from ten to twenty minutes. I avoid the use of all escharotics and antiseptics before introducing the filling. The delicate pulp tissue must suffer severe injury from such violent agents as pure carbolic acid, creosote, etc., etc., which many dentists are in the habit of using. I allow the oxychloride to remain for a year. I then remove it and insert gold, or whatever other permanent filling material I may see fit. I have invariably found the pulps alive and healthy, and protected by a layer of osteo-dentine. I perform this operation on all classes of patients, and at all seasons of the year. Of course the rapidity of the healing and deposition of osteo-dentine depends somewhat upon the health and temperament of the patient.

*Dr. Walker.*—I tried this method fifteen years ago; in some cases I had beautiful results, in very many, however, I failed. I attributed this lack of success to the influence of the atmosphere in London. In the same way surgical operations in large cities are not as successful as in small ones.

*Dr. De Trey.*—I have not found that the atmosphere made any difference as to the success of the operation. There was no rubber dam at the time Dr. Walker performed this operation, and I do not believe that it can be properly done without it.

*Dr. Sachs.*—Do you perform this operation for patients who are living at a distance, and who must leave the next day?

*Dr. De Trey.*—Yes. I never hesitate to resect and cap a pulp, even if the patient is leaving half an hour afterwards.

*Dr. Sachs.*—I divide exposed pulps into three classes: 1st, those exposed by accident; 2d, those exposed by caries, not inflamed; 3d, those exposed by caries, and inflamed. In the third class I never hesitate to use arsenic. I have tried amputating the pulp and in forty per cent have had bad results, while with the proper use of arsenious acid we may reduce the failures to one per cent. I use cotton and creosote, or carbolic acid for filling the roots, a method which has given me much greater satisfaction than either gutta percha or oxychloride of zinc.

*Dr. De Trey.*—I never use arsenic in my practice; when I find it absolutely necessary to devitalize a pulp I prefer pure creosote; with a fine broach I can work this up into the canals around the nerve, and at the first sitting frequently remove the greater portion, with very little pain to the patient.

*Dr. Patton.*—I use arsenic and find even then that two or three applications may be necessary before the pulp is entirely devitalized. Dr. De Trey's patients must be different from those in Cologne.

*Dr. Cunningham.*—I have tried Dr. De Trey's method, but have not had equal success, though Dr. De Trey is undoubtedly right in his assertion that many pulps are killed by over-treatment. In some cases I do not hesitate to remove the softened dentine over the pulp, and expose and resect a portion of it with the engine burr. I believe with Dr. Walker that bad drainage has a very ill effect upon the treatment of dental pulps.

*Dr. Patton.*—I object to the term resection; it is at best only scratching, and when performed with the burr it is still worse. I should like to see some one make a clean cut of a dental pulp with an engine burr.

*Dr. Jenkins.*—There is no doubt that various results are obtained from the same method of treatment by different operators, and with different patients.

Dr. De Trey's wonderfully delicate manipulation is one to which but few dentists are able to attain. I believe too, that nationality makes a very great difference in the susceptibility of the dental pulp to conservative treatment. With patients of Slavonic origin we may attempt operations with a fair chance of success, which would be sure failures with patients of Teutonic blood. For many years it was my custom to attempt the preservation of the vitality of nearly all pulps which came under my care, and many cases which I believed to have succeeded turned out in later years to be anything but fortunate; in other instances the apparent success of the treatment resulted only from a mummification of the pulp under a filling of oxychloride of zinc. I now never attempt, for Saxons, to cap anything but a freshly exposed pulp, and even then there is no certainty that the operation will succeed.

Dr. Cunningham, of Cambridge, England, read an article entitled "A System of Dental Notation; being a Code of Symbols for the Use of Dentists in recording Surgery Work." The design of the paper was to suggest and advocate a system of notation which should be adapted to universal use, and by means of which members of the profession could, by means of a few symbols, communicate to each other the operations which had been performed in any mouth, and the condition of the teeth at the time of performing the operations. A committee of three was appointed to report on this subject at the next annual meeting.

Dr. Miller, of Berlin, read an article of some length on the "Etiology of Dental Caries." This paper will appear in the INDEPENDENT PRACTITIONER.

The society accepted Dr. Miller's views of dental caries without a dissenting voice.

The Secretary read a paper by Dr. Blount, of Geneva, on "The Causes of the Failure of Gold as a Filling Material." (See page 588).

DISCUSSION.

*Dr. De Trey.*—I believe that heavy gold makes a better surface than any other kind. I use No. 240 for this purpose; it must of course be used judiciously, and be packed with smooth points.

*Dr. Elliott.*—I look upon the filling of teeth as the most important part of dentistry, and any improvements in the methods of inserting fillings, in instruments for filling, or in any filling materials, should be considered as of the greatest value.

It was formerly thought that solidity was no necessary element in a good filling; that a filling might be so soft as to be easily penetrated by a fine excavator, and still preserve the tooth for an indefinite number of years; that a filling could be as soft as cork and still be efficient. We now consider uniform density throughout the filling as one of the most important features of a gold plug. It must moreover be solid from the foundation, for unless this precaution is taken, and every piece thoroughly condensed before the following one is inserted, it will not be possible by any amount of malleting on the surface of the filling to produce a good result. The character of the operation which we perform must also be largely determined by the condition and structure of the tooth.

There is only one way left for us, and that is to be eclectic in our practice. I now use a combination of tin and gold in nearly one-third of all the cavities that I fill. My use of hard gold is confined to fairly assessible cavities, and I would for my own part rather have the distal surfaces of bicuspids and molars filled with amalgam than with cohesive gold.

*Dr. Galbraith.*—I have not been long in practice, and yet I have seen enough to convince me that hard gold is by no means necessary for beautiful contour filling. Some of the finest and most durable work of this nature that I have ever seen has been done with soft gold, and with combinations of tin and gold.

*Dr. Walker.*—In the London Dental Hospital we have previously used cohesive gold exclusively; we now use both cohesive and non-cohesive, and we find that students can make fillings of non-cohesive gold in from two to three hours, which require from five to six with the electric mallet. I am convinced by observations in the hospital as well as in my own practice, that it is not justifiable to restrict ourselves to the use of cohesive gold alone. I frequently line cavities with soft gold and complete with hard, always preparing my gold on the day of use.

*Dr. Elliott.*—I am Examiner in the London Dental Hospital with which Dr. Walker is connected, and I have been surprised and pleased to observe the excellence of the operations performed by the students. This skill in operating is accompanied, as you all know, by a most thorough theoretical knowledge. I have myself made different medical examinations. The easiest of these was that for M. D., at the University of New York. My subsequent examination for D. D. S. at the Philadelphia Dental College amounted to absolutely nothing, and I am convinced that the English system of dental education, combining as it does thoroughness both in theory and practice, must in the long run triumph over the American system.

*Dr. Cunningham.*—Americans in Europe have fallen into disrepute with some of their colleagues in America, because they are said to have become eclectic in their practice, or in other words to have retrograded. This was the highest compliment that could have been paid them, although not intended.

A great deal has been said about noble and base materials, but we know no base materials in dentistry. The material becomes base only when it is put to improper use, and I have certainly seen most debasing effects from the injudicious use of gold in places where material which the operator would have scorned to use might have effected the salvation of the tooth. The views of many advocates of hard gold are narrow and confined, and designed to do great harm.

No dentist of an unbiased mind should allow himself to be led to such unreasonable extremes. No more can I adopt the creed of those advocates of the New Departure who would exclude gold entirely from their practice. I need gold, amalgam, gutta percha, cement, etc., etc., nor could I for a moment think of excluding one of them without doing great injustice to myself and to my patients.

*Dr. Jenkins.*—I have great compassion for any man, and for any man's patients who is confined to any one particular method of practice. We are dentists, not for the purpose of running a hobby, but to render the greatest possible service to our patients, and that we can only do by availing ourselves of every possible means at hand.

As for the New Departurists, their practice is in general good, but the principles upon which it was originally based were long ago proven, by a member of our society, to be utterly groundless. We are not new departurists, nor hard-goldists, nor amalgamists, but *dentists.* As far as the beauty of the operation is concerned, it is absolutely impossible after years to tell which are the fillings made of soft foil, which of hard foil, and which of combined foil.

*Dr. Bishop.*—I was at first very much prejudiced against amalgam. I then used soft gold, and have fillings thirty-five years old still in perfect condition. When hard gold was introduced I advocated it zealously for a short time, but found out its weak points before I had gone far enough to do any very great damage.

I now begin nearly all fillings with soft gold. I do not hesitate to use amalgam in large cavities difficult of access.

(To be continued.)

## Figures and Tables

**Figure f1:**